# Response to: Aspirin for patients after TIPS. An old dog with new tricks?

**DOI:** 10.1007/s12072-022-10394-9

**Published:** 2022-07-29

**Authors:** Leon Louis Seifert, Philipp Schindler, Dominik Bettinger, Jonel Trebicka, Moritz Wildgruber, Hauke Heinzow

**Affiliations:** 1 Medical Clinic B, Department of Gastroenterology, Hepatology, Endocrinology, Infectiology, University Hospital Muenster, 48149 Muenster, Germany; 2 Clinic for Radiology, University Hospital Muenster, 48149 Muenster, Germany; 3 Department of Medicine II, Medical Center University of Freiburg, University of Freiburg, 79106 Freiburg, Germany; 4 Department of Radiology, University Hospital LMU Munich, 81377 Munich, Germany; 5 Medical Clinic I, Klinikum Der Barmherzigen Brüder Trier, 54292 Trier, Germany

## To the Editor

We would like to thank Dr. Wang, Dr. Qi and their team for their interest and appreciate the raised issues regarding our study entitled ‘Aspirin improves transplant-free survival after TIPS implantation in patients with refractory ascites—a retrospective multicenter cohort study’ [1].

Despite the retrospective character of our work and the extensive discussion of accompanying limitations, the focused and detailed TIPS programs of our centers reduce potential bias of the presented results.

While liver-specific mortality is important to report, unfortunately we do not have this information for all patients. Yet, all-cause mortality is at least as important, since recently we demonstrated that cirrhosis also as comorbidity multiplies mortality rate [2].

Another important point raised by Wang et al. is the indication of aspirin. However, as recently also in the Baveno VII guidelines is clearly stated that aspirin is beneficial and should be continued in patients with an indication for its use. The same is true for the use of other disease modifying drugs such as statins and non-resorbable antibiotics [3]. As we already have discussed this topic we cannot exclude any overlap of those drugs with aspirin, but still the use of aspirin was independently associated with outcome. On the other hand, the use of non-selective beta blockers (NSBBs) is usually discontinued after successful TIPS implantation, and therefore a potential bias due to their use is unlikely.

A further criticism by Wang et al. is the use propensity score matching (PSM) resulting in a smaller sample size after matching associated with possible type I errors. Although we agree that this may occur, the reduction of sample size is a result of the application of robust matching criteria and an acceptable caliper width. The created cohort, matched using age, sex, MELD-score and platelet count to be able to compare patients with or without aspirin administration after TIPS, is well balanced and without significant differences in variables influencing prognosis in cirrhotic patients.

In the context of treatment safety it is imperative to keep in mind that the imbalance in hemostasis does not only put cirrhotic patients at risk concerning bleeding events but also thrombotic events [4]. In our opinion, there are no data supporting a clear contraindication for use of aspirin in cirrhotic patients. At our institutions, aspirin was only administered in patients with a platelet count > 50,000/µl.

Our data provide first data on a potentially beneficial effect of aspirin in cirrhotic patients receiving TIPS. Nonetheless it is important to underline that our data are insufficient to support a strong clinical recommendation but it encourages prospective studies.
